# High Affinity Binding of N2-Modified Guanine Derivatives Significantly Disrupts the Ligand Binding Pocket of the Guanine Riboswitch

**DOI:** 10.3390/molecules25102295

**Published:** 2020-05-13

**Authors:** Michal M. Matyjasik, Simone D. Hall, Robert T. Batey

**Affiliations:** Department of Biochemistry, University of Colorado, Boulder, CO 80309, USA; Michal.Matyjasik@colorado.edu (M.M.M.); Simone.Hall@colorado.edu (S.D.H.)

**Keywords:** guanine riboswitch, aptamer, RNA, x-ray crystallography, isothermal titration calorimetry, transcription

## Abstract

Riboswitches are important model systems for the development of approaches to search for RNA-targeting therapeutics. A principal challenge in finding compounds that target riboswitches is that the effector ligand is typically almost completely encapsulated by the RNA, which severely limits the chemical space that can be explored. Efforts to find compounds that bind the guanine/adenine class of riboswitches with a high affinity have in part focused on purines modified at the C6 and C2 positions. These studies have revealed compounds that have low to sub-micromolar affinity and, in a few cases, have antimicrobial activity. To further understand how these compounds interact with the guanine riboswitch, we have performed an integrated structural and functional analysis of representative guanine derivatives with modifications at the C8, C6 and C2 positions. Our data indicate that while modifications of guanine at the C6 position are generally unfavorable, modifications at the C8 and C2 positions yield compounds that rival guanine with respect to binding affinity. Surprisingly, C2-modified guanines such as *N*2-acetylguanine completely disrupt a key Watson–Crick pairing interaction between the ligand and RNA. These compounds, which also modulate transcriptional termination as efficiently as guanine, open up a significant new chemical space of guanine modifications in the search for antimicrobial agents that target purine riboswitches.

## 1. Introduction

Since the discovery of riboswitches, RNA elements have been considered a new potential route to develop small-molecule antimicrobial therapeutics [1,2,3]. Several important reasons underscore this rationale. First, riboswitches are naturally predisposed to binding small molecules. The upstream region of a riboswitch is the aptamer domain, a highly structured sequence generally found in bacterial mRNA leader sequences, that specifically binds a small organic metabolite or cation [4,5]. The occupancy status of the aptamer domain is communicated to a downstream expression platform that directs expression machinery. Thus, chemical analogs of the cognate metabolite might serve as potent downregulators of genes essential for housekeeping or virulence that are controlled by riboswitches [1]. Second, while riboswitches are broadly distributed throughout bacteria, including many of medical interest, they are not observed in humans [6,7]. This suggests that orthogonal compounds could be found that specifically target these RNAs without interacting with human biological functions. Third, RNA is a well-proven target of successful therapeutics, particularly several classes of compounds that bind ribosomal RNAs with a high affinity and specificity [8]. Finally, there has been a resurgent interest in targeting a number of diseases through their RNA targets [8,9]. The difficulty in targeting a specific RNA element with a small molecule has prompted efforts to use structurally characterized riboswitches as learning tools for understanding the binding of small molecules to RNA, since the mode of binding is known at the molecular level and can be explored using medicinal chemistry approaches. An example of such an effort using the flavin mononucleotide (FMN) riboswitch was the development of the antimicrobial agent 5FDQD [10,11].

**Figure 1:**
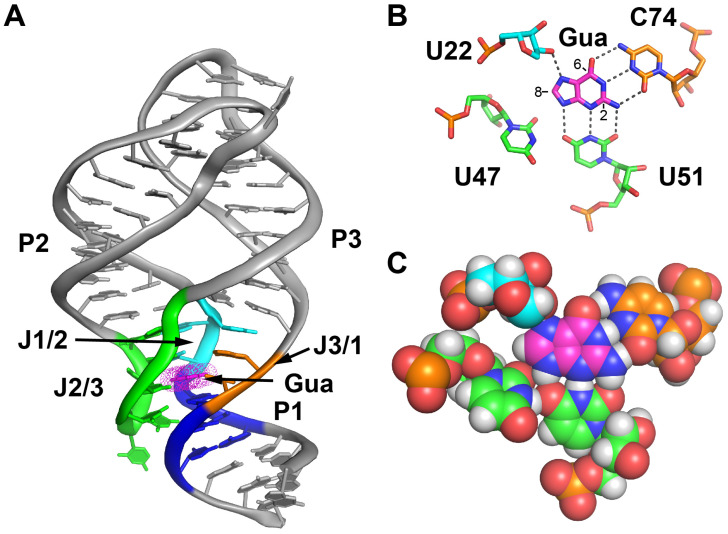
Structure of the guanine-guanine riboswitch (GR) complex (**A**) Ribbon cartoon representation of the global structure of the ligand-RNA complex, with key regions in the ligand binding pocket highlighted in color (helix P1, blue; J1/2, cyan; J2/3, green; J3/1, orange). Guanine is shown in magenta. The same coloring scheme is used throughout. This is from PDB 1Y27 [12]. (**B**) Recognition of guanine by the ligand binding pocket situated in the three-way junction. U51 and C74 form three-hydrogen bond pairing interactions with the guanine ligand. The C8, C6 and C2 positions are denoted on the ligand. (**C**) The same perspective as in panel B, with a Van der Waals spheres to emphasize the space adjacent to the C8, C6 and C2 positions that serves as the starting point for exploring modifications. Hydrogen atoms have been added to fully illustrate the hydrogen bonding interactions.

The purine riboswitch family, and particularly its guanine/adenine binding class, has been a powerful model system for understanding small molecule-RNA interactions and approaches to drugging RNAs. This family of riboswitches, united by a common secondary and tertiary structure and a pattern of conservation of nucleotide identity, is broadly distributed across bacteria and highly prevalent in Firmicutes, including medically important pathogens [7,13,14,15]. Structural and biochemical analyses of the aptamer domain of several representative members, including the guanine riboswitch that regulates the *xpt-pbuX* transcriptional unit in *Bacillus subtilis,* has revealed a binding pocket that is almost completely solvent inaccessible and forms hydrogen bonds with all of the ligand’s polar groups (Figure 1A) [12,16,17]. Discrimination between guanine and adenine is conferred by the pyrimidine residue at position 74 (nucleotide numbering used throughout this work is that of the *B. subtilis xpt* guanine riboswitch) via the Watson–Crick base pairing with the ligand. Discrimination between nucleobases (guanine and adenine) and nucleosides (2′-deoxyguanosine) is primarily dictated by the identity of the pyrimidine residue at position 51—a uridine (U) for nucleobase recognition and cytidine (C) for nucleoside recognition [18,19,20]. Across a broad spectrum of compounds that bind the guanine riboswitch, the positions of the nucleotides contacting the ligand are fixed (Figure 1B), although a small displacement of cytidine 74 towards the minor groove in relation to the ligand has been observed for a few C6-modified guanine derivatives such as *O*6-methylguanine and 2-aminopurine [21].

The earliest efforts to target the purine riboswitch with alternative small molecules focused upon pyrimidines. Based upon crystal structures of the bound guanine and adenine riboswitch aptamer domains, it was proposed that a spectrum of pyrimidine residues could bind to both the adenine riboswitch and the guanine riboswitch bearing a C74U mutation that confers adenine selectivity. Several tri- and tetra-amino pyrimidine compounds were shown to bind purine riboswitches with low- to mid-micromolar affinities [22]. Extending on this observation, several pyrimidine compounds were identified that bind to guanine riboswitches with low micromolar affinities and exhibited antimicrobial activity against *Staphylococcus aureus* by blocking *guaA* expression [23]. While one of these compounds, PC1, performed only modestly in reducing infection in a bovine model, this work demonstrated the potential for targeting riboswitches as a promising route to novel antimicrobial therapeutics [24].

Other efforts to identify compounds that productively bind purine riboswitches have taken two routes. The first used the crystal structure of the C74U mutant of the *B. subtilis xpt-pbuX* guanine aptamer to virtually screen for novel compounds that bind adenine-responsive riboswitches [25]. This yielded several compounds that interact with the RNA similarly to adenine, but all bound with very modest affinities (mid- to high-micromolar). While these lead compounds were not suitable to pursue as potential antimicrobials, they may be further improved using medicinal chemistry approaches.

The second route took a structure-based approach to designing guanine analogs that could potentially bind the guanine riboswitch aptamer domain [26,27]. Both studies focused upon modifications of the C2 and C6 positions of guanine, hypothesizing that functional groups at these positions would minimally perturb the guanine-bound aptamer structure. The first survey found that a number of C2- and C6-modified guanine analogs bind to the *B. subtilis xpt-pbuX* guanine riboswitch with nanomolar affinity using an in-line probing assay, but that only one, 6-*N*-hydroxylaminopurine, showed any potential to inhibit bacterial growth [26]. However, a subsequent analysis of 6-*N*-hyroxylaminopurine antimicrobial activity suggested that inhibition of the guanine riboswitch was not the mechanism [28]. A more comprehensive survey of C2 and C6-modified purines identified further analogs that are able to bind to the *C. difficile guaA* guanine riboswitch, a potential target of antimicrobial therapeutics [23], with affinities in the low-micromolar to high-nanomolar range [27]. However, a strong correlation was not observed between high affinity binding (K_D_) and *C. difficile* growth inhibition (MIC). Instead, modest improvements to growth inhibition were made through lipophilic modifications, suggesting that improvement in the pharmacokinetic properties of these compounds, such as cellular uptake, is critical for increasing the efficacy of these guanine analogs.

The above findings highlight the difficulties in developing practical RNA-targeting therapeutics using structure-based approaches. While structure-based approaches using computational and medicinal chemical approaches have identified a spectrum of compounds that bind the guanine riboswitch with high affinities, these compounds did not have the requisite pharmacokinetic (PK) properties to act upon the target riboswitch in vivo. Indeed, the most successful RNA-targeting drugs—including the FMN riboswitch-targeting compound ribocil—have been found using phenotypic screens rather than design approaches [8]. However, from a pharmacodynamics (PD) perspective, it is still important to understand the routes to increasing a compound’s affinity for its target, which is correlated to efficacy. In addition, for riboswitches, the compound must be able to modulate its regulatory activity, thereby mimicking the natural effector [1]. Together, being able to effectively model the PK/PD relationship is a central aspect of any drug discovery effort [29].

In this study, we have examined a set of guanine compounds modified at the C8, C6 or C2 positions to better understand their effect on binding affinity, RNA structure and in vitro regulatory activity in order to obtain a more complete understanding of their pharmacodynamic properties. One of the most surprising results from previous structure-function design efforts was that C2-modified guanine analogs, such as *N*2-acetylguanine (molecular structures used in this study are given in Figure S1), bind the guanine riboswitch with nanomolar affinity. This is unexpected, as modifications at this position would present significant steric challenges to either U51 or C74 in the guanine-bound structure (Figure 1C). The structural analysis of a complex between the aptamer domain of the *B. subtilis xpt-pbuX* guanine riboswitch (referred to as GR in this work) and *N*2-acetylguanine reveals that this compound dramatically remodels the binding pocket by expulsion of the discriminator nucleotide C74, making the bound ligand far more solvent accessible. This compound, along with several other C2-modified guanine analogs, bind the riboswitch with affinities that rival guanine and are able to effectively regulate transcriptional termination. Together, these results suggest the C2 position is the most promising site for further medicinal chemistry efforts to find a compound that targets purine riboswitches.

## 2. Results

### 2.1. C8-Modified Guanine Derivatives Minimally Perturb the Binding Pocket

The ability of C8-substituted purine analogs to bind to purine riboswitches has not been explored extensively. The initial biochemical characterization of the guanine riboswitch found that 8-methylxanthine binds with ~2000-fold lower affinity than xanthine (100 µM and 50 nM, respectively) [14], and 8-chloroadenine was found to be unable to bind the adenine riboswitch [15]. The crystal structures of the guanine and adenine riboswitches reveal that the ligand’s C8 is less than 3.5 Å distance from three nucleotides (A21, U22 and A47), suggesting that there is insufficient room in the purine ligand bound binding pocket to accommodate any functional group at this site. Thus, C8 was considered to be unsuitable for modification of purine analogs that could bind GR with high affinity [26,27].

To more thoroughly test this position, we examined two compounds that have sterically small modifications at this site, 8-aminoguanine and 8-hydroxyguanine, by isothermal titration calorimetry (ITC), which is considered the gold-standard technique for quantifying binding affinities [30,31]. Surprisingly, the addition of an amino group at the C8 position has only a modest penalty for binding GR (K_rel_ = 17, Table 1) and binds with an affinity that is higher than one of its biological effectors, hypoxanthine (K_D_ of 36 ± 13 and 240 ± 30 nM, respectively) (representative thermograms are given in Figure S2). The penalty for this same modification to hypoxanthine is a higher (58-fold, Table 1), but an 8-amino group does not completely abrogate binding. 8-hydroxyguanine has an even higher affinity for GR, with a K_D_ of 10 ± 4 nM (K_rel_ = 5_,_ Table 1), again suggesting that sterically modest functional groups at the 8-position are minimally disruptive to binding.

To determine how the 8-amino modification of guanine is accommodated within the binding pocket, we determined the crystal structure of the 8-aminoguanine-GR complex. To make a direct comparison between guanine- and 8-aminoguanine-bound GR, we determined the crystal structure of the guanine-GR complex to a 1.6 Å resolution (crystallographic data collection and model statistics are given in Supplementary Table S1), which is a higher resolution than the previously determined analogous complex at 2.4 Å (PDB 1Y27) [12]. As expected, this structure showed no significant differences with a 1.3 Å resolution structure of the same RNA bound to hypoxanthine (PDB 4FE5) crystallized under similar conditions [32].

**Figure 2:**
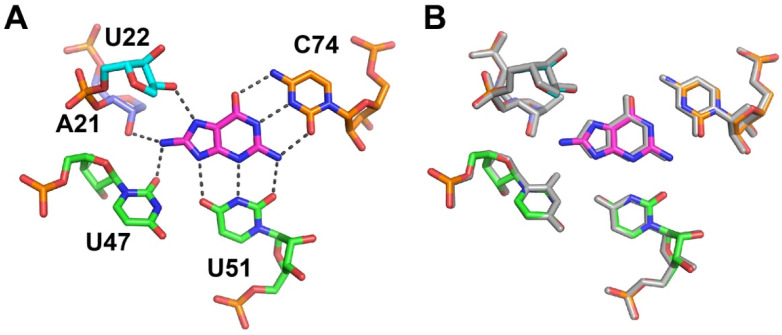
Recognition of 8-aminoguanine by GR. (**A**) Hydrogen bonding interactions between 8-aminoguanine (magenta) and GR. Additional hydrogen bonding interactions are observed between N8 of the ligand and the A21 2′-hydroxyl group and U47 O2 carbonyl oxygen of GR. (**B**) Superimposition of the 8-aminoguanine-GR binding pocket with the guanine-GR complex (grey).

The crystal structure of the 8-aminoguanine-GR complex revealed a set of ligand–RNA interactions that is identical with that of guanine (Figure 1B), with the exception that the 8-amino group forms two additional hydrogen bonds with A21 O2’ and U47 O2 (Figure 2A). Further, the presence of the 8-amino group is minimally disruptive to the local structure in the binding pocket. The superposition of nucleotides around the binding pocket (nts 20–24, 46–53 and 73–76) and the ligand reveals that 8-aminoguanine and adjacent residue C74 are displaced by ~0.3 Å (Figure 2B), only slightly greater than the maximum likelihood coordinate errors in each structure (0.16 and 0.24 Å, Table S1). The only significant differences between the two binding pockets is that the O2 of U47 is 3.0 Å from the N9 of guanine and 3.6 Å from the equivalent position of 8-aminoguanine; additionally, the distance between the A21 O2’ and C8 of the ligand increases from 3.7 Å for guanine to 4.2 Å for 8-aminoguanine (Figure 2A,B). This indicates that there is a small displacement of atoms in GR around the 8-amino group to accommodate this additional moiety. Thus, the two structures are essentially equivalent except for the slight perturbations caused by the 8-amino group, which are partially compensated for by additional productive hydrogen bonds.

### 2.2. C6-Modified Guanine Derivatives Shift C74 Towards the Minor Groove

The binding of guanine analogs that bear modifications at the *O*6 position have been extensively examined across multiple studies. For example, the methylation of *O*6 has a significant energetic penalty for binding the guanine riboswitch as compared to guanine [14,21], which was confirmed in this study (K_rel_ = 3100, Table 1). A crystal structure of a *O*6-methylguanine-GR complex revealed that the methyl group causes C74 to shift towards the minor groove, establishing two hydrogen bonds between the Watson–Crick face of *O*6-methylguanine and C74 [21]. However, in that same study it was observed that a bulkier C6 modification, *O*6-benzylguanine, abrogates binding. In contrast, several studies in which the C6 position was modified with *N*-linked functional groups indicate that a spectrum of purine analogs bind the guanine riboswitch with sub-micromolar affinities [26,27]. These data motivated us to further examine a set of guanine derivatives to firmly establish their binding characteristics.

The binding of several guanine derivatives containing bulky C6 substituents indicated that they weakly bind the guanine riboswitch. Using ITC, we measured the affinities for *O*6-methylcyclohexylguanine (also known as NU2058, a kinase inhibitor [33]) as ~8100-fold weaker than guanine and *N*6-cyclopropyl-9H-purine-2,6-diamine as ~12,000-fold weaker than guanine, corresponding to K_D_ values above 10 µM (Table 1). These affinities are only modestly weaker than *O*6-methylguanine, suggesting that the additional functional groups do not interact substantially with the RNA. Consistent with previous observations, *O*6-benzylguanine does not detectably bind GR. This suggests that the rigid aromatic ring of *O*6-benzylguanine clashes with the RNA, while the more flexible non-aromatic rings can be accommodated.

**Figure 3:**
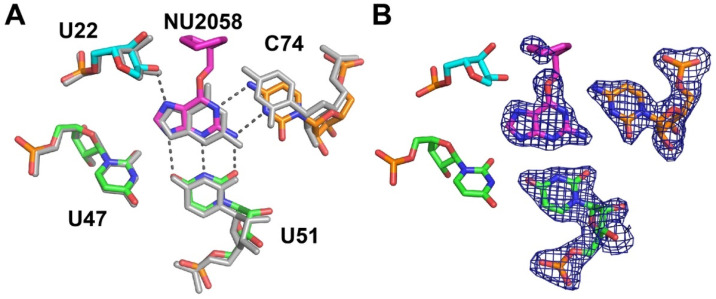
Recognition of NU2058 by GR. (**A**) Hydrogen bonding interactions between NU2058 and GR. The binding pocket of the guanine-GR complex is superimposed, emphasizing the shift of C74 towards the minor groove, resulting in two hydrogen bonds between the ligand and RNA. (**B**) Binding pocket of the NU2058-GR complex with density from a simulated annealing composite map contoured at 1 around the ligand and nucleotides U51 and C74. Only two atoms in the methylcyclohexyl group have a clear density, indicating that this moiety of the ligand is highly conformationally flexible.

The structure of the *O*6-methylcyclohexylguanine-GR complex was determined using x-ray crystallography to examine the binding pocket’s ability to accommodate a bulkier O6 substitution. The superimposition of the binding pocket and ligand with that of the guanine-GR complex reveals the same shift of C74 towards the minor groove relative to the ligand, as previously observed for *O*6-methylguanine (Figure 3A). However, there is no strong electron density corresponding to the cyclohexyl group (Figure 3B), indicating that this moiety has significant conformational freedom within the complex and likely does not strongly interact with the RNA. This is consistent with the ITC binding data (Table 1). The fitting of this moiety into the structure indicates that the cyclohexyl ring is adjacent to several phosphate groups. This suggests that the improvement in affinity might be gained by the addition of hydrogen bonding groups to the cyclohexyl ring. Given the numerous polar functional groups in this region of the RNA, there is substantial opportunity to form productive interactions with complimentary C6 modifications. However, these favorable interactions would have to overcome a substantial energetic penalty that is associated with the minor groove shift of C74 [21].

### 2.3. C2-Modified Guanine Derivatives Displace C74 from the Binding Pocket

Like modifications at the C6 positions, C2-modified purine derivatives have been investigated previously in several studies. A number of derivatives were found to bind GR with a high affinity, including *N*2-acetylguanine with a reported K_D_ value of 0.5 nM, as determined by in-line probing, which is ~10-fold lower than the literature ITC K_D_ value of guanine [26]. A more detailed structure-activity study examined the binding of a large set of C2-modified purines to the *C. difficile guaA* guanine riboswitch and found a diverse set of compounds that can bind the aptamer with affinities in the low micromolar range [27]. These observations raise a question about how C2-modified purine derivatives can productively bind guanine riboswitches without altering the local architecture of the binding pocket. In the guanine-bound form, both hydrogens of the N2-amino group engage in hydrogen bonding interactions with carbonyl oxygens of U51 and C74; modifications at the C2 position would require the disruption of interactions with one of the pyrimidine residues. Thus, it would be expected that either U51 or C74 would have to be displaced.

To validate the above findings, we measured the binding of a set of C2-modified guanine derivatives to GR using ITC. *N*2-acetylguanine binds GR with a K_D_ of 300 ± 10 nM, which is ~three-fold lower than the observed K_D_ of 1.05 µM for the *C. difficile* riboswitch. The addition of two methyl groups to the acetyl group yields a compound, *N*2-isobutyrylguanine, that is able to bind GR with an affinity that rivals guanine (K_D_ = 7.4 ± 0.5 nM and 2.1 ± 0.7 nM, respectively; Table 1). The addition of a third methyl group to the analog in *N*2-pivaloylguanine resulted in a significant loss of binding affinity. However, larger functional groups at the C2 position do not preclude high affinity binding; *N*2-phenoxyacetyl guanine also bound with a low nanomolar affinity (8.8 ± 0.5 nM, Table 1). In contrast to C2 modifications that acylate the exocyclic amine, the simple methylation of N2 as found in *N*2-methylguanine binds very poorly to GR (17 ± 1 µM, Table 1), suggesting that the acylation of the amino group is a highly favorable modification. A more extreme modification to the Watson–Crick face of guanine is found in 4,6,7,8-tetrahydro-8-hydroxy-6-methylprimido[1,2-a]purin-10(3H)-one, which creates a six-member ring by connecting the N1 and C2 positions of the purine ring (Figure S1). This compound binds GR with an affinity of 79 ± 1 µM, which is significantly weaker than most C2-modified analogs, but reinforces the finding that the compounds that would cause a steric clash with C74 in the biologically relevant guanine-GR complex are still capable of being recognized. The importance of ligand hydrogen bonding to U51 is reinforced by the observation of no detectible binding of the related compound pyrimido[1,2-a]purin-10(1H)-one, which cannot form three hydrogen bonds with this nucleotide.

**Figure 4:**
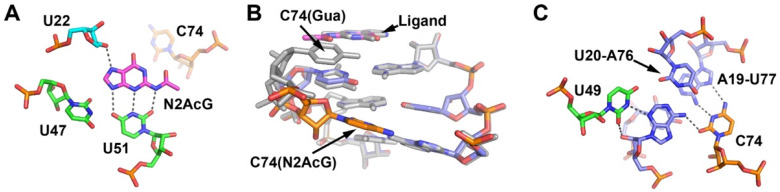
Crystal structure of the *N*2-acetylguanine-GR complex. (**A**) Hydrogen bonding interactions between the ligand and GR. (**B**) Superimposition of the *N*2-acetylguanine-GR complex with the guanine-GR complex (grey). The base of nucleotide C74 is stacked between nucleotides 73 and 75 in the guanine-GR complex, whereas in the *N*2-acetylguanine-GR complex the base of C74 is situated in the major groove adjacent to the base pairs A19-U77 and U20-A76 in the P1 helix. (**C**) Hydrogen bonding interactions between C74 and two base pairs in the P1 helix.

The crystal structure of the *N*2-acetylguanine-GR complex reveals that C2-modified purines significantly perturb the local binding pocket. The crystal structure of GR bound to guanine and *N*2-acetylguanine superimpose very well (r.m.s.d. = 0.33 Å), indicating that this analog does not disrupt the global architecture. However, the superimposition of the local binding pockets reveals that the presence of the *N*2-acetyl group results in the expulsion of C74 from the coaxial stacking arrangement of nucleotides in P3, J3/1 and P1 (Figure 4A). Instead of engaging the Watson–Crick face of the ligand, as is observed in all ligand-bound structures of aptamers in the purine riboswitch family, C74 is positioned within the major groove of the P1 helix and makes hydrogen bonding contacts to the U20-A76 and A19-U77 base pairs (the second and third junction-proximal base pairs in the P1 helix) (Figure 4B,C).

### 2.4. Cytosine at Position 74 Is Required for High Affinity C2-Modifed Purine Binding

Since C74 is expelled from the P3-J3/1-P1 helical stack, we asked whether other nucleotide identities at this position could support the binding of C2-modified guanine derivatives. *N*2-phenoxyacetyl guanine was used in these experiments, since it displays a substantially higher affinity for GR RNA than *N*2-acetylguanine. The C74U substitution, which abrogates guanine binding [34], binds *N*2-phenoxyacetyl guanine with a K_D_ value of 7.5 ± 0.8 µM (Table 2), indicating that this compound is less sensitive to the identity of position 74. This also would suggest that a C2-modified guanine derivative may be able to productively bind adenine-responsive purine riboswitches that naturally have the uridine at the 74 position. Interestingly, in a prior study of the interaction of purine analogs with the GR(C74U) mutant, it was observed that the analogs did not induce a minor groove shift with uridine as they did with cytosine [21]. These data suggest that in the context of GR, a uridine at position 74 has a greater energetic penalty to adopt the alternative conformation that is expelled from the P3-J3/1-P1 nucleotide stack. Purines at the 74 position are even more deleterious to ligand binding (C74A and C74G, Table 2). Thus, while nucleotide 74 is expelled from the P3-J3/1-P1 coaxial stack and no longer directly interacts with the ligand, cytidine at this position strongly promotes the binding of *N*2-modified purine analogs.

### 2.5. The Third Junction-Roximal Base Pair in P1 Influences Binding Affinity of an N2-Modified Guanine Analog

In the crystal structure of the *N*2-acetylguanine-GR complex, C74 is spatially adjacent to the second and third junction-proximal base pairs in P1. While the second pair (U20-A76) forms a minor groove base triple with U49 in J2/3 in response to ligand binding, the third pair (nts 19 and 77) is only phylogenetically conserved, with a preference for a R-Y pair [6,7,14]. Thus, we determined whether the identity of the 19–77 base pair influences the *N*2-phenoxyacetyl guanine binding affinity via its ability to form favorable interactions with C74. The other Watson–Crick pairs and a G19-U77 wobble have only a modest contribution to ligand binding; the most favorable base pair at this position is a C19-G77 pair, which binds *N*2-phenoxyacetyl guanine ~four-fold more tightly than the GR wild-type A19-U77 pair (Table 2). In the context of a C74U substitution, the identity of the 19–77 pair also has a small influence on ligand binding affinity, again with the C19-G77 pair being most favorable (Table 2). Thus, the interaction between nucleotide 74 and the 19–77 base pair in P1 has only a modest effect on ligand binding.

The comparison of the binding of guanine and two C2 derivatives to the GR(A19C-U77G) mutant RNA shows that guanine remains the highest affinity compound. By ITC, guanine binds GR(A19C-U77G) RNA with a K_D_ of 0.85 ± 0.31 nM, a ~2.5-fold higher affinity than the wild-type GR. This indicates that while the third junction-proximal base pair in the P1 helix is not directly involved in ligand recognition, it has a small influence on affinity (Table 3). *N*2-isobutyrylguanine has an observed K_D_ of 4.5 ± 2.0 nM, a ~1.6-fold higher affinity than to wild-type GR, which is a ~5.3-fold weaker binding than guanine to GR(A19C-U77G) RNA (Table 3). Likewise, *N*2-phenoxyacetylguanine binds GR(A19C-U77G) RNA ~2.6-fold more weakly than guanine (Table 3).

### 2.6. Combined C2 and C6 Modifications Are Tolerated

There are many guanine analogs that bear multiple substitutions that could potentially bind purine riboswitches. One example is *O*6-methylcyclohexyl-*N*2-benzenesulfonamidoguanine (NU6102), a C2-modified variant of NU2058 that is as a potent kinase inhibitor [33]. Notably, NU6102 binds GR with a K_D_ of 5.6 ± 0.6 µM, which is a three-fold higher affinity than NU2058 (Table 1). Thus, the C2 modification of NU6102 plays a productive role in the binding affinity that modestly compensates for the unfavorable *O*6-methylcyclohexyl modification of NU2058. Despite lacking the *N*2-amide moiety that appears to promote GR binding affinity, the *N*2-benzenesulfonamido group alone may support higher binding affinity, like the *N*2-phenoxyacetyl group.

### 2.7. Modified Purines Support Regulatory Switching

**Figure 5:**
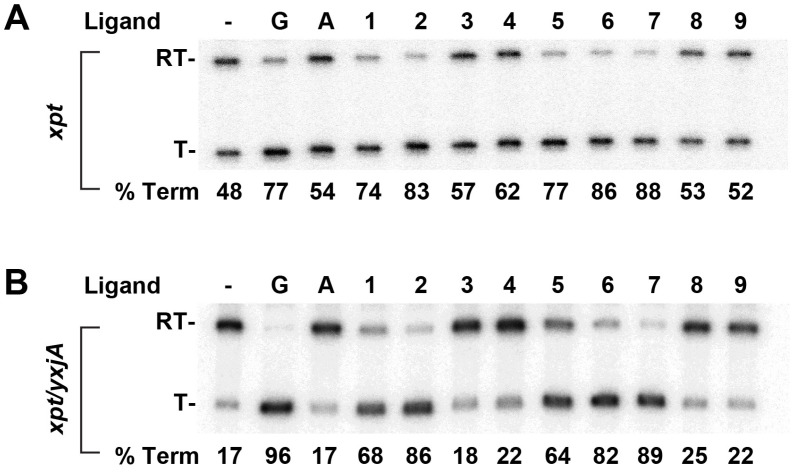
Single-turnover transcription assays using select guanine analogs. (**A**) Transcription termination assays using the wild type *B. subtilis xpt-pbuX* guanine riboswitch. The ^32^P-labeled RNA bands in the denaturing polyacrylamide gel are denoted as “RT” for read-through full length RNA and “T” for terminated RNA. As an “OFF” switch, the guanine riboswitch terminates transcription in the presence of 20 µM guanine (compare the no ligand (−) and guanine (G) lanes); the % termination product is given below each lane. All the reactions (except the no ligand control) have a 20 µM compound. Lanes: (−), no ligand; G, guanine; A, adenine; 1, 8-aminoguanine; 2, *O*6-methylguanine; 3, *O*6-benzylguanine; 4, NU2058; 5, *N*2-acetylguanine; 6, *N*2-phenoxyacetyl guanine; 7, N2-isobutyrylguanine; 8, *N*2-pivaloylguanine; 9, 4,6,7,8-tetrahydro-8-hydroxy-6-methylpyrimido[1,2-a]purin-10(3H)-one. (**B**) The same compounds as in panel A, tested against the *xpt*/*yxjA* chimeric riboswitch.

The above results illustrate that purine modifications at the C2 position are mostly likely to result in analogs with affinities for GR that rival guanine. However, we questioned whether the significant remodeling of the binding pocket via the expulsion of C74 would still be conducive to the ligand-dependent secondary structure switching by the expression platform. To address this, we used a single-turnover transcription assay to examine the potential for these compounds to regulate gene expression [35]. The *B. subtilis xpt-pbuX* guanine riboswitch is an “OFF” switch in which ligand binding promotes the formation of an intrinsic terminator, causing RNA polymerase to disengage from mRNA synthesis [14]. Single turnover transcription assays were performed using a DNA template that placed the *xpt-pbuX* transcriptional unit under the control of a strong constitutive promoter. The products of the transcription were resolved by denaturing polyacrylamide gel electrophoresis for quantification [36,37,38]. The comparison of the transcription of the *xpt* gene in the absence and presence of 20 µM guanine reveals a modest ability to repress read-through transcription (48% and 77% terminated product, respectively; Figure 5A). This observation is consistent with the modest levels of regulatory control by this riboswitch in *B. subtilis* [39]. As a control, adenine, which does not bind the guanine riboswitch, does not promote termination (Figure 5A).

The analysis of a subset of C8-, C6- and C2-modified guanine analogs to promote transcriptional termination at a 20 µM concentration revealed that modifications at any of the three positions support regulatory activity (Figure 5A). Notably, multiple C2-modified guanine analogs were able to terminate transcription similarly to guanine, with two, *N*2-phenoxyacetyl guanine and *N*2-isobutyrylguanine, performing almost as well as guanine. Further, the trends in termination activity correlate with the measured binding affinities. Because of the modest degree of transcriptional termination observed for the wild-type *xpt* riboswitch, we also tested this panel of compounds against a more robust guanine-responsive riboswitch that is a fusion of the aptamer domain of the *B. subtilis xpt* guanine riboswitch and the expression platform of the *B. subtilis yxjA* guanine riboswitch. This chimera, based upon a “mix-and-match” strategy [40], responds to guanine with a significantly greater dynamic range (17% to 96% termination, respectively; Figure 5B). Importantly, this synthetic riboswitch shows the same patterns of responsiveness to analogs as the wild type guanine riboswitch. Again, the high affinity *N*2-phenoxyacetyl guanine and *N*2-isobutyrylguanine compounds show a very high regulatory activity. These data are consistent with previous studies showing that select C2- and C6-modified purines are capable of repressing reporter gene expression and cell growth [26,27]. Importantly, the trends in regulatory activity strongly correlate with binding affinity, indicating that, from a pharmacodynamic perspective, affinity improvements translate into an increase in transcriptional termination efficacy.

## 3. Discussion

This study sought to provide a structural interpretation of results from several surveys of guanine analogs that bind the guanine riboswitch as well as examine a set of compounds not studied in previous work. While prior studies have focused both on the modifications of guanine at the C6 and C2 positions, our results reveal that the latter position is a much more favorable site for further exploration of chemical space. This is because of the unexpected observation that compounds that sterically clash with the guanine-bound position of C74 cause its expulsion from the GR binding pocket P3-J3/1-P1 stack. The disruption of the binding pocket at C74, surprisingly, has only a modest energetic penalty that can be presumably overcome by new favorable interactions with the RNA, as in *N*2-phenoxyacetyl guanine and *N*2-isobutyrylguanine. Neither of these functional groups is expected to form interactions with the RNA beyond energetically modest van der Waals interactions. Thus, designing molecules that are capable of hydrogen bonding or forming electrostatic interactions with the surrounding RNA may yield compounds that have higher affinities for GR than guanine.

An additional route to higher affinity compounds may be to combine C8 and C2 modifications. Hydroxy- and amino-modification at the C8 position are capable of forming additional productive hydrogen bonding interactions with the RNA, as revealed by the 8-aminoguanine-GR complex crystal structure. Since the modest energetic penalty of these compounds as compared to guanine may arise from a small displacement of C74, these modifications in combination with a C2 modification that completely displaces C74 could be another route to compounds that have higher affinities than guanine. The ability of a second modification to facilitate binding is exemplified by NU6102, whose C2 modification overcomes some of the energetic penalty of the *O*6-methylcyclohexyl group.

The structure of the *N2*-acetylguanine-GR complex also has implications for the proposed mechanism of ligand binding by purine riboswitches. Since the bound ligand is almost completely encapsulated by the RNA, a central question is what conformational changes are associated with binding. An early proposal suggested that C74 along with the J3/1 region of the binding pocket forms the structurally rigid region of the binding pocket that initially docks with the ligand, and J1/2 and J2/3 then subsequently collapse to yield the crystallographically observed ligand-RNA complex [34,41]. This model was supported by structural studies of the homologous *Vibrio vulnificus add* adenine riboswitch (AR) using x-ray free-electron laser crystallography, which enabled the visualization of both an apo and intermediate-bound state of the RNA. The structures of the AR binding-competent apo state (apo2) and the intermediate bound state indicate that U74 plays a pivotal role in ligand recognition, with J1/2 and J2/3 being flexible [42,43].

The structure of the *N*2-acetylguanine-GR complex challenges this view. Since at no point in the binding process is an *N*2-modified ligand expected to productively engage with C74, the above cannot be the only binding mechanism. Instead, these data argue that there may be multiple pathways to form the crystallographically observed ligand-GR complex. In the case of guanine and adenine binding, the above pathway that is supported by a wealth of biochemical and structural data is likely highly populated. *N*2-acetylguanine and other C2-modified ligands, on the other hand, may exploit a different pathway. An explicit solvent molecular dynamics simulation of AR yielded a model in which a dynamic J2/3, and U51 in particular, forms an initial encounter complex with the ligand, which is then brought into the binding pocket to engage U74 [44]. C2-modified ligands could still form the initial encounter complex with U51 in the same fashion as adenine/guanine, but upon docking with the core binding pocket would either actively displace C74 or only fully engage when C74 transiently unstacks from the adjacent nucleotides. Since the C/U74 and U51 initial engagement models of binding have many other features in common, such as the high degree of mobility of the J2/3 region of the junction, it is not inconceivable that both mechanisms are used. Indeed, being able to exploit a greater range of conformers within the ensemble of apo-states may substantially facilitate complex formation at rates compatible with the kinetically-controlled mechanisms of riboswitches [45,46].

A common theme amongst riboswitches is that the cognate ligand is almost always fully encapsulated with the aptamer domain [4]. This substantially restricts the chemical space that can be explored in structure-guided efforts to discover effective antimicrobial therapeutics. However, the observation that some C2-modified compounds can displace a critical nucleotide in the guanine riboswitch with surprisingly little energetic penalty may be relevant to other riboswitches. While high-throughput phenotypic screens that have been used to find compounds with antimicrobial properties that target riboswitches have been successfully implemented [47,48], many compounds with favorable pharmacodynamics but unfavorable pharmacokinetics will have failed to be observed. In this light, it is important to develop high throughput in vitro screening approaches for riboswitches to more fully explore unexpected chemical space.

## 4. Materials and Methods

### 4.1. Synthesis of RNA for ITC and X-ray Crystallography

All the RNA sequences used in this work are listed in Table S2. DNA oligonucleotides encoding *B. subtilis xpt* guanine riboswitch (GR) aptamer sequences were obtained from Integrated DNA Technologies (IDT) (Coralville, IA, USA) with standard desalting. The RNA was prepared from PCR-amplified DNA templates by in vitro transcription with T7 RNA polymerase using standard approaches [49]. For x-ray crystallography, the transcription template used to synthesize RNA had two 2′-*O*-methoxy groups at the 3′-end to prevent the addition of untemplated nucleotides by T7 RNA polymerase [50,51]. After completion, the transcription reaction was buffer exchanged into Milli-Q water and purified using denaturing polyacrylamide gel electrophoresis. The product RNA was extracted using the crush and soak technique in 0.5× Tris-EDTA (TE) buffer (5 mM Tris, 0.5 mM ethylenediaminetetraacetic acid (EDTA), pH 8.0) and concentrated to 500 µL using a 10 kDa Amicon centrifugal microconcentrator. For ITC, the transcription reactions were exchanged into the ITC buffer (50 mM K-HEPES, 100 mM KCl, 20 mM MgCl_2_, pH 7.5) and concentrated to 500 µL using a 10 kDa Amicon centrifugal microconcentrator with no further purification. The RNA concentrations were determined using calculated extinction coefficients at 260 nm and stored at −20 °C.

### 4.2. Crystallization of GR RNA-Ligand Complexes

The *xpt* GR aptamer domain RNA in complex with various small molecule ligands was crystallized at ambient room temperature (24 °C) using the hanging drop diffusion method. The RNA-ligand complexes were prepared as solutions containing 250 μM RNA and 500 μM ligand in 0.5x TE buffer. To crystallize the complex, drops composed of 2 µL RNA-ligand mixture and 2 µL precipitant mixture (10 mM potassium 4-(2-hydroxyethyl)-1-piperazineethanesulfonic acid (HEPES) pH 7.5, 20–23% *v*/*v* PEG 3000, 30–50 mM cobalt hexammine and 600–650 mM ammonium acetate) were suspended above a reservoir solution of 500 μL of 35% 2-methyl-2,4-pentanediol (MPD). Initial crystals were small needles, which were used to microseed new drops to yield bigger single crystals. Hanging drops were incubated for 1–3 weeks to allow the crystals to reach maximum size. To cryoprotect, the samples were soaked in a cryoprotectant (precipitant mixture with MPD increased to 25%) for 5 min and flash frozen in liquid nitrogen. The data were collected on a home-source Rigaku MicroMax-007 HF X-ray source with a Dectris Pilatus 3R 200K-A detector, then indexed, integrated and scaled with HKL3000 [52].

### 4.3. Structure Determination and Model Refinement

For the models of GR7 in complex with guanine and 8-aminoguanine, an electron density map was calculated using a molecular replacement with Phaser in PHENIX using the GR RNA as a starting model (PDB ID 4FE5) [53]. To improve the core nucleotide and ligand positioning accuracy and prevent model bias, cobalt hexammine and acetate molecules were built into the electron density followed by refinement, further improving the phasing. The ligand density was then unambiguously clear, allowing for the precise positioning of the respective ligand. The map and model were refined and the solvent was built until a maximum agreement between the map and the model was reached. During this process, to mitigate the effect of model bias, multiple rounds of combined high temperature (5000 K) simulated annealing and maximum-likelihood refinement were performed [54].

For the models of GR7 in complex with N2-acetylguanine and O6-cyclohexylmethyl guanine, an electron density map was calculated by molecular replacement in PHENIX using the GR RNA as a starting model with nucleotides 51 and 74 excluded (PDB ID 4FE5) [53]. Cobalt hexammine and acetate molecules were built into the electron density after molecular replacement followed by refinement to further improve the phasing. At this stage, the electron density was very well-defined to build positions 51 and 74, followed by refinement to improve the phasing before ligand building. The entire ligand density was then clear (for *N*2-acetylguanine) and the ligand nucleobase and C6 methylene group density was clear (for *O*6-cyclohexylmethylguanine) to position each respective ligand. The map and model were refined and the solvent was built until a maximum agreement between the map and the model was reached.

### 4.4. Isothermal Titration Calorimetry

Isothermal titration calorimetry was performed using previously described methods [21,55]. The ligands were dissolved in ITC buffer, heated at 37 °C for 15 min, and filtered using Costar centrifuge tube 0.2 micron filters. Depending on the binding affinity and ligand solubility, the experiments were either carried out by injecting ligand (70–5000 µM) into an RNA-filled sample cell (5–50 µM) or by performing a reverse titration where RNA (55–910 µM) was injected into a ligand filled sample cell (3–95 µM). All the titrations were performed at 25 °C using a MicroCal ITC_200_ and collected in triplicate. The *c* value for all experiments were between 0.1 and 4300, a range that enables the accurate determination of binding affinities [56]. The data were fit to a single-site binding model using Origin 7 ITC software (MicroCal Software).

### 4.5. Single-Turnover In Vitro Transcription Assays

Transcription assays were performed using both wild-type *B. subtilis xpt* and chimeric *xpt*/*yxjA* riboswitches [40,57,58]. DNA templates were prepared following previously published methodology (DNA sequences used for the single turnover transcription assays given in Table S3) [57]. Briefly, the templates for wild-type riboswitches were synthesized using PCR amplification from *B. subtilis* genomic DNA. Recursive PCR, with overlapping oligonucleotides of the *xpt* aptamer domain and *yxjA* expression platform, was used to make the chimeric riboswitch template [59]. The T7A1 promoter was placed immediately upstream of the transcription start site in all the DNA templates, allowing for an efficient initiation with *E. coli* RNA polymerase. The DNA templates were purified using the Omega Bio-tek E.N.Z.M.A cycle pure kit and were sequence verified prior to use in the transcription assays.

The regulatory activity of wild-type and chimeric riboswitches were determined using an in vitro transcription assay following previously described methods [57]. The transcription assays were performed in a 25 µL reaction volume. For every reaction, 17 µL containing 12.5 µL of 2x transcription buffer (140 mM Tris-Cl, pH 8.0, 140 mM NaCl, 0.2 mM EDTA, pH 8.0, 28 mM β-mercaptoethanol, and 70 µg/mL BSA), 50 ng DNA template, 2.5 µL of 25 µM MgCl_2_, 0.5 µCi α-[^32^P]-ATP and 0.25 units of *E. coli* RNA polymerase σ^70^ holoenzyme (New England Biolabs) were prepared. These solutions were heated for 10 min at 37 °C. The transcription was initiated by the addition of 8 µL of a solution containing 62.5 µM ligand, 312.5 µM ribonucleotide triphosphates and 0.2 mg/mL heparin, followed by incubation for another 20 min at 37 °C. The reactions were quenched by adding 25 µL of a denaturing loading dye (95% *v*/*v* formamide, 10 mM EDTA pH 8.0, 0.25% *w*/*v* bromophenol blue, 0.25% *w*/*v* xylene cyanol), followed by heating at 65 °C for 3 min. The products were separated using urea-polyacrylamide gel electrophoresis on a 6% 29: 1 denaturing acrylamide: bisacrylamide gel, dried and exposed overnight to a phosphor screen. The band intensities were quantified using ImageJ. The percent terminated was calculated by dividing the terminated product intensity by that of the sum of the read through and terminated intensities. All the gels were replicated in triplicate and the error in percent terminated was reported as the standard deviation of the mean.

## Figures and Tables

**Table 1:** Isothermal titration calorimetry (ITC) measurements of ligand binding to GR RNA.

Ligand	*n*	K_D_ (nM)	K_rel_^1^	∆H (kJ/mol)	c Value
guanine	1.0 ± 0.1	2.1 ± 0.7	1	−190 ± 10	1400 ± 300
hypoxanthine	1.1 ± 0.1	240 ± 30	120	−120 ± 10	180 ± 10
**C8-Modified Ligands**					
8-aminohypoxanthine	1.0 ± 0.1	14,000 ± 1000	6600	−290 ± 10	0.37 ± 0.02
8-aminoguanine	1.2 ± 0.4	36 ± 13	17	−70 ± 10	930 ± 370
8-hydroxyguanine	1.1 ± 0.1	10 ± 4	5.0	−110 ± 10	1900 ± 600
**C6-Modified Ligands**					
6-chloroguanine	0.90 ± 0.02	340 ± 30	170	−120 ± 10	130 ± 20
*O*6-methylguanine	0.82 ± 0.10	6400 ± 400	3100	−120 ± 10	9.0 ± 1.9
*N*6-cyclopropyl-9H-purine-2,6-diamine	1.1 ± 0.1	25,000 ± 3000	12,000	−110 ± 10	2.0 ± 0.2
*O*6-benzylguanine	NA	No binding	NA	NA	NA
NU2058	1.2 ± 0.1	17,000 ± 1000	8000	−2200 ± 600	0.31 ± 0.01
**C2-Modified Ligands**					
*N*2-acetylguanine	1.1 ± 0.1	300 ± 10	140	−160 ± 10	100 ± 10
*N*2-isobutyrylguanine	1.1 ± 0.1	7.4 ± 0.5	3.6	−210 ± 10	4300 ± 300
*N*2-pivaloylguanine	0.97 ± 0.05	230,000 ± 10,000	110,000	−1300 ± 100	0.11 ± 0.01
*N*2-methylguanine	0.99 ± 0.03	17,000 ± 1000	8300	−100 ± 10	5.2 ± 0.2
*N*2-phenoxyacetyl guanine	0.80 ± 0.06	8.8 ± 0.5	4.3	−180 ± 10	1700 ± 100
4,6,7,8-tetrahydro-8-hydroxy-6-methylprimido[1,2-a]purin-10(3H)-one	1.0 ± 0.2	79,000 ± 1000	38,000	−58 ± 8	0.25 ± 0.01
pyrimido[1,2-a]purin-10(1H)-one	NA	No binding	NA	NA	NA
**C2-,C6-Modified Ligand**					
NU6102	1.1 ± 0.1	5600 ± 600	2700	−100 ± 10	1.2 ± 0.3

^1^ K_rel_ = K_D, ligand_/K_D, guanine._

**Table 2:** ITC of *N*2-phenoxyacetyl guanine with mutants of GR RNA.

Mutant	*n*	K_D_ (nM)	K_rel_^1^	∆H (kJ/mol)	*c* Value
WT	0.80 ± 0.06	8.8 ± 0.5	1	−180 ± 10	1700 ± 100
C74U	1.2 ± 0.3	7500 ± 800	850	−96 ± 27	3.8 ± 0.4
C74G	1.0 ± 0.2	42,000 ± 2000	4800	−110 ± 90	1.8 ± 0.2
C74A	1.0 ± 0.1	85,000 ± 25,000	9700	−25 ± 3	0.36 ± 0.09
A19C U77G	1.3 ± 0.2	2.2 ± 0.6	0.25	−110 ± 20	3200 ± 1000
A19G U77C	0.99 ± 0.02	4.9 ± 1.2	0.56	−180 ± 10	3600 ± 1000
A19G U77	1.0 ± 0.1	5.4 ± 1.2	0.61	−190 ± 10	2800 ± 700
A19U U77A	1.0 ± 0.1	7.8 ± 1.6	0.89	−170 ± 10	2300 ± 400
A19C C74U U77G	1.1 ± 0.1	4100 ± 400	470	−190 ± 10	7.5 ± 0.5
A19G C74U U77C	1.0 ± 0.1	44,000 ± 4000	5000	−150 ± 10	0.50 ± 0.04
A19U C74U U77A	1.0 ± 0.1	66,000 ± 5000	7500	−120 ± 10	0.46 ± 0.03

^1^ K_rel_ = K_D, Mut-N2PAcG_/K_D, WT-N2PAcG._

**Table 3:** ITC measurements of ligand binding to GR(A19C-U77G).

Ligand	*n*	K_D_ (nM)	K_rel_^1^	∆H (kJ/mol)	*c* Value
guanine	1.0 ± 0.1	0.85 ± 0.31	1	−130 ± 10	5700 ± 1800
*N*2-isobutrylguanine	0.98 ± 0.03	4.5 ± 2.0	5.3	−130 ± 10	2600 ± 900
*N*2-phenoxyacetyl guanine	1.3 ± 0.2	2.2 ± 0.6	2.6	−110 ± 20	3200 ± 1000

^1^ K_rel_ = K_D, GR(A19C-U77G)-ligand_/K_D, GR(A19C-U77G)-guanine._

## Supplementary Materials

The following are available online. Figure S1: Chemical structures of compounds used in this study; Figure S2: Representative ITC thermograms of wild type GR-ligand titrations; Figure S3: Representative ITC thermograms of mutant GR-ligand titrations; Table S1: crystallographic data and refinement statistics; Table S2: RNA sequences used in this study; Table S3: DNA sequences used for single turnover transcription assays.
