# Supplementary material for: High Affinity Binding of N2-Modified Guanine Derivatives Significantly Disrupts the Ligand Binding Pocket of the Guanine Riboswitch
Source: Molecules. 2020 May 13;25(10):2295. doi: 10.3390/molecules25102295 (PMC7287874; doi:10.3390/molecules25102295)
Supplement: Supplementary file 1 [file molecules-25-02295-s001.pdf]

molecules

MDPI

Supplemental Information
High Affinity Binding of N2-Modified Guanine Derivatives Significantly Disrupts the Ligand Binding Pocket of the Guanine Riboswitch

Michal M. Matyjasik †, Simone D. Hall † and Robert T. Batey *

Department of Biochemistry, University of Colorado, Boulder, Colorado, CO 80309, USA; Michal.Matyjasik@colorado.edu (M.M.M.); Simone.Hall@colorado.edu (S.D.H.); robert.batey@colorado.edu (R.T.B.)

* Correspondence: robert.batey@colorado.edu; Tel.: +1-303-7352159

† These authors contributed equally to this work.

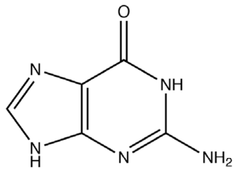

guanine

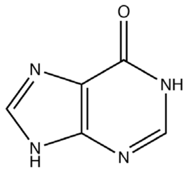

hypoxanthine

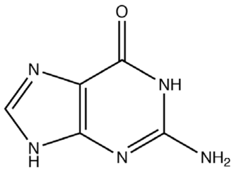

adenine

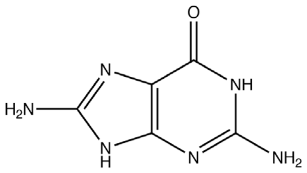

8-aminoguanine

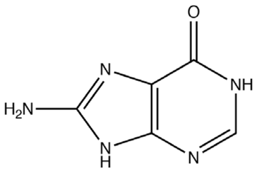

8-aminohypoxanthine

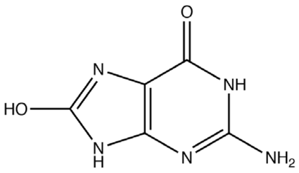

8-hydroxyguanine

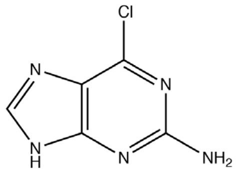

6-chloroguanine

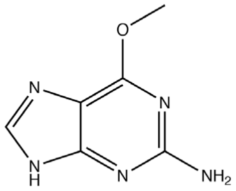

*O*6-methylguanine

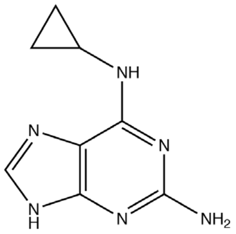

*N*6-cyclopropyl-9H-purine-2,6-diamine

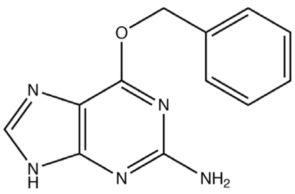

O6-benzylguanine

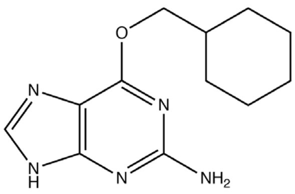

O6-methylcyclohexyl-guanine (NU2058)

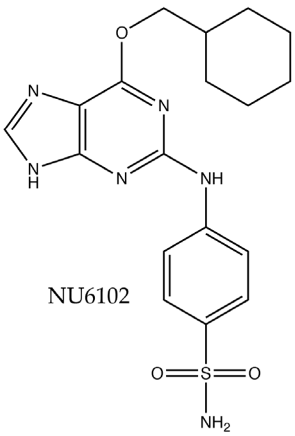

NU6102

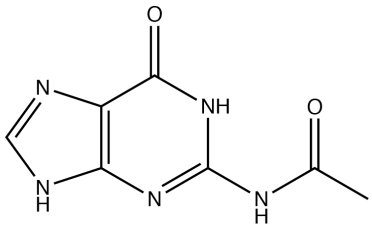

*N*2-acetylguanine

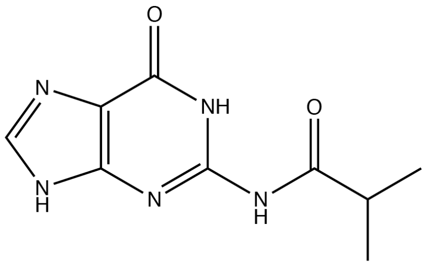

*N*2-isobutyrylguanine

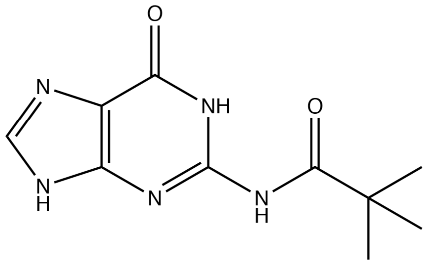

*N*2-pivaloylguanine

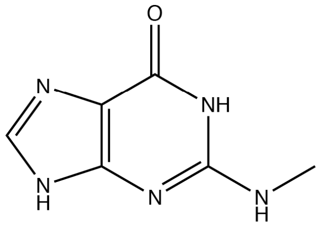

*N*2-methylguanine

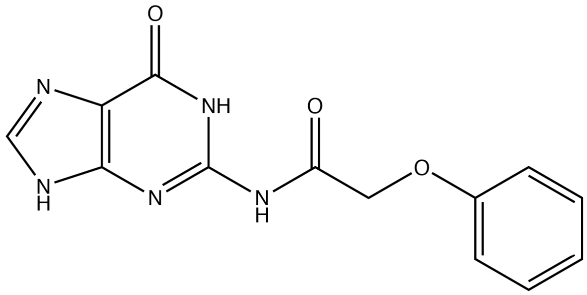

*N*2-phenoxyacetyl guanine

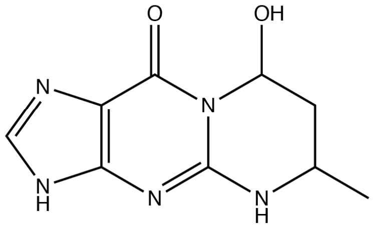

4,6,7,8-tetrahydro-8-hydroxy-6-methylprimido[1,2-a]purin-10(3H)-one

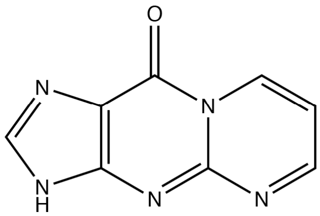

pyrimido[1,2-a]purin-10(1 H)-one

Figure S1. Chemical structures of compounds used in this study.

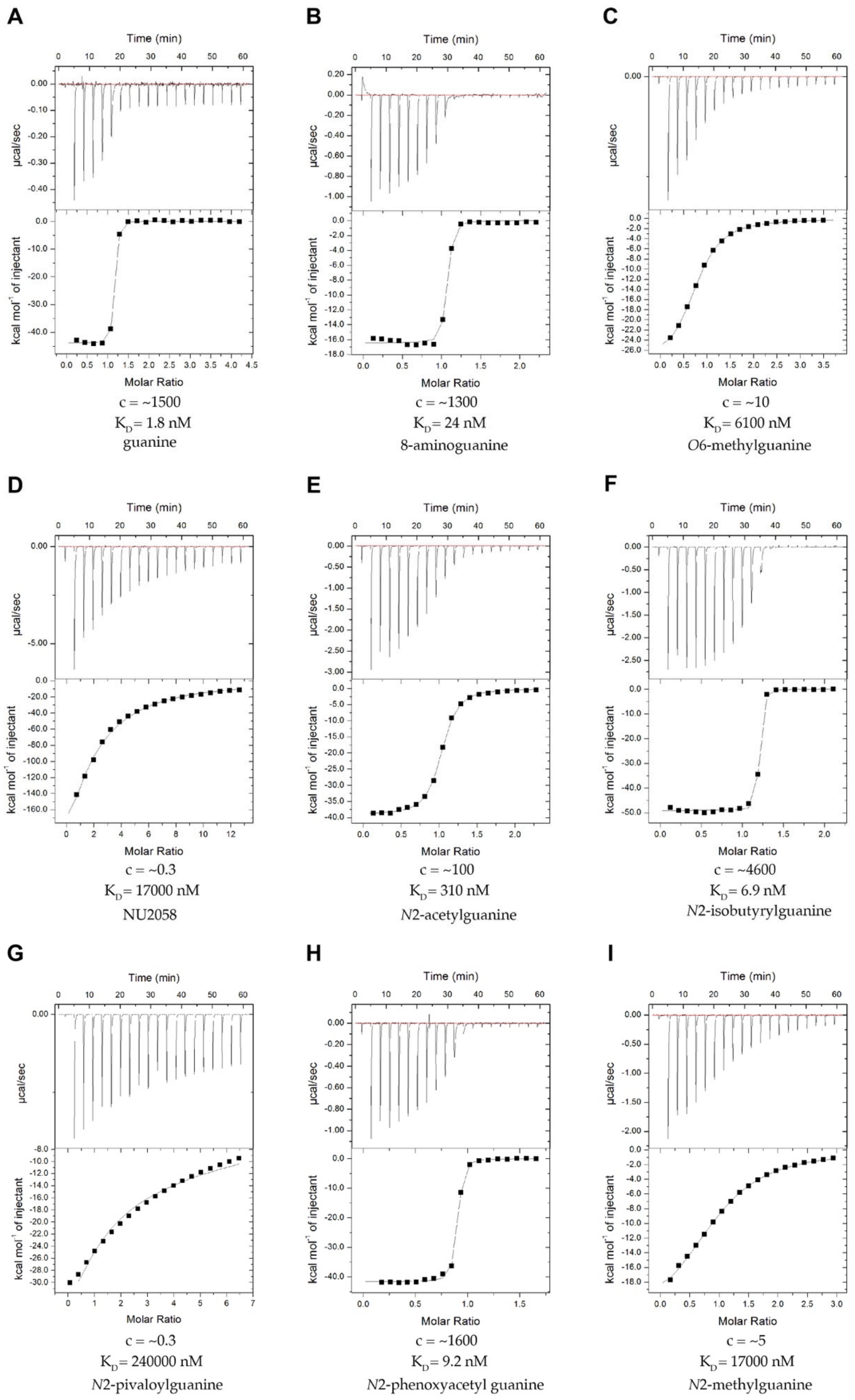

Figure S2. (**A**)–(**I**) Representative ITC thermograms for wild type GR-ligand titrations.

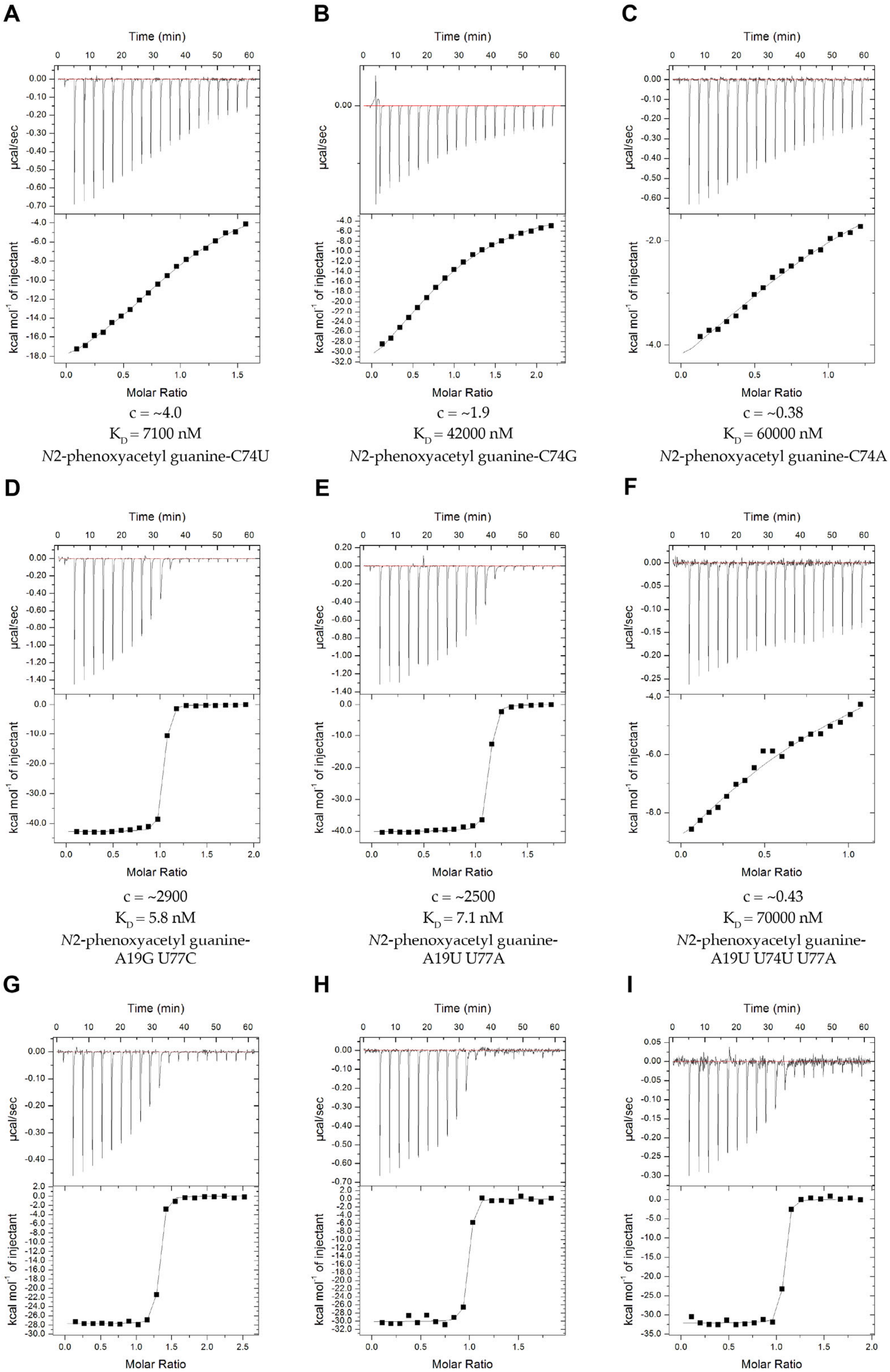

(A) c = ~4.0, K_D = 7100 nM, *N*2-phenoxyacetyl guanine-C74U

(B) c = ~1.9, K_D = 42000 nM, *N*2-phenoxyacetyl guanine-C74G

(C) c = ~0.38, K_D = 60000 nM, *N*2-phenoxyacetyl guanine-C74A

(D) c = ~2900, K_D = 5.8 nM, *N*2-phenoxyacetyl guanine-A19G U77C

(E) c = ~2500, K_D = 7.1 nM, *N*2-phenoxyacetyl guanine-A19U U77A

(F) c = ~0.43, K_D = 70000 nM, *N*2-phenoxyacetyl guanine-A19U U74U U77A

(G) c = ~2100, K_D = 2.8 nM, *N*2-phenoxyacetyl guanine-A19C U77G

(H) c = ~2800, K_D = 4.0 nM, *N*2-isobutyrylguanine-A19C U77G

(I) c = ~3900, K_D = 1.2 nM, guanine-A19C U77G

Figure S3. (**A**)–(**I**) Representative ITC thermograms for mutant GR-ligand titrations.

Table S1. Crystallographic data collection and model refinement statistics.

GR with Ligand (PDB ID)	Guanine (6UBU)	8-Aminoguanine (6UC8)	*O*6-Methylcyclohexyl Guanine (6UC9)	*N*2-Acetylguanine (6UC7)
Data Collection				
Space group	C121	C121	C121	C121
Cell dimensions				
a, b, c (Å)	132.18, 35.26, 42.28	132.55, 35.18, 41.93	133.47, 35.17, 41.89	132.11, 35.08, 41.61
α, β, γ (°)	90, 90.86, 90	90, 90.35, 90	90 91.554 90	90, 90.99, 90
Wavelength (Å)	1.5406	1.5406	1.5406	1.5406
Resolution[a]	19.66 - 1.60 (1.65 - 1.60)	19.96 - 1.90 (1.97 - 1.90)	19.86 - 1.94 (2.01 - 1.94)	19.74 – 1.80 (1.86 – 1.80)
R_{sym}	0.131 (0.361)	0.073 (0.228)	0.158 (0.445)	0.079 (0.182)
$I / \sigma I$	27.25 (3.37)	16.75 (1.51)	11.96 (2.09)	19.30 (3.05)
CC (1/2)	0.872 (0.622)	0.955 (0.900)	0.897 (0.588)	0.969 (0.903)
Completeness (%)	83.41 (23.15)	87.57 (36.89)	97.91 (85.26)	93.83 (56.89)
Redundancy	4.5 (1.0)	3.7 (1.5)	6.6 (3.8)	3.0 (1.9)
Refinement				
Resolution	19.66 - 1.60 (1.62 - 1.60)	19.96 - 1.90 (1.94 - 1.90)	19.86 – 1.94 (1.98 – 1.94)	19.74 - 1.80 (1.83-1.80)
Number of unique reflections	21872 (593)	13646 (572)	14355 (1226)	16940 (1007)
R_{work}	0.198 (0.393)	0.187 (0.248)	0.187 (0.255)	0.184 (0.219)
R_{free}	0.220 (0.505)	0.224 (0.329)	0.211 (0.315)	0.212 (0.268)
Number of atoms				
RNA	1422	1422	1422	1422
ligand	72	72	71	70
ions/solvent	240	231	197	240
B-factors				
RNA	27.96	27.78	29.22	27.56
ligand	25.75	27.26	29.86	30.78
ions/solvent	53.44	28.36	29.80	29.14
R.m.s. deviation				
Bond lengths (Å)	0.005	0.005	0.005	0.005
Bond angles (°)	1.05	1.01	1.04	1.05
Coordinate error (Å)[b]	0.16	0.24	0.24	0.17

[a] Values in parenthesis represents the highest resolution shell. [b] Maximum likelihood based.

Table S2. RNA sequences used in this study.

GR RNA	Sequence
wild type	GGACAUAUAAUCGCGUGGAUAUGGCACGCAAGUUUCUACCGGGCACCGUAAAUGUCCGACUAUGUCCA
C74U	GGACAUAUAAUCGCGUGGAUAUGGCACGCAAGUUUCUACCGGGCACCGUAAAUGUCCGAUUAUGUCCA
C74G	GGACAUAUAAUCGCGUGGAUAUGGCACGCAAGUUUCUACCGGGCACCGUAAAUGUCCGAGUAUGUCCA
C74A	GGACAUAUAAUCGCGUGGAUAUGGCACGCAAGUUUCUACCGGGCACCGUAAAUGUCCGAAUAUGUCCA

A19C<br>U77G	GGACCUAUAAUCGCGUGGAUAUGGCACGCAAGUUUCUACCGGGCACCGUAAAUGUC<br>CGACUAGGUCCA
A19G<br>U77C	GGACGUAUAAUCGCGUGGAUAUGGCACGCAAGUUUCUACCGGGCACCGUAAAUGU<br>CCGACUACGUCCA
A19G	GGACGUAUAAUCGCGUGGAUAUGGCACGCAAGUUUCUACCGGGCACCGUAAAUGU<br>CCGACUAUGUCCA
A19U<br>U77A	GGACUUAUAAUCGCGUGGAUAUGGCACGCAAGUUUCUACCGGGCACCGUAAAUGU<br>CCGACUAAGUCCA
A19C<br>C74U<br>U77G	GGACCUAUAAUCGCGUGGAUAUGGCACGCAAGUUUCUACCGGGCACCGUAAAUGUC<br>CGAUUAGGUCCA
A19G<br>C74U<br>U77C	GGACGUAUAAUCGCGUGGAUAUGGCACGCAAGUUUCUACCGGGCACCGUAAAUGU<br>CCGAUUACGUCCA
A19U<br>C74U<br>U77A	GGACUUAUAAUCGCGUGGAUAUGGCACGCAAGUUUCUACCGGGCACCGUAAAUGU<br>CCGAUUAAGUCCA

Table S3. DNA sequences used for single turnover transcription assays.

DNA	Sequence
5′ primer	GCG CTA GCC ACA GCT AAC AC
3′ primer	TAT TAG AAT TCG GTA CCC GGG GAT CCT CTA GAG TC
xpt wild type	GCGCTAGCCACAGCTAACACCACGTCGTCCCTATCTGCTGCCCTAGGTCTATGA<br>GTGGTTGCTGGATAACTTTACGGGCATGCATAAGGCTCGTATAATATATTCAAT<br>TAAATAGACGTCTAGGAACACTCATATAATCGCGTGGATATGGCACGCAAGTTT<br>CTACCGGGCACCGTAAATGTCCGACTATGGGTGAGCAATGGAACCGCACGTGT<br>ACGGTTTTTTGTGATATCAGCATTGCTTGCTCTTTATTTGAGCGGGCAATGCTTTT<br>TTTATTACTAGTACATTTAAGTAAAGGAGTTTGTTATGACCATGATTACGCCAA<br>GCTTGCATGCCTGCAGGTCGACTCTAGAGGATCCCCGGGTACCGAATTCTAATA
xpt/*yxjA* chimera	GCGCTAGCCACAGCTAACACCACGTCGTCCCTATCTGCTGCCCTAGGTCTATGA<br>GTGGTTGCTGGATAACTTTACGGGCATGCATAAGGCTCGTATAATATATTCAAT<br>TAAATAGACGTCATCTTAGAAAAAGACATTCTTGTATAATCGCGTGGATATGGC<br>ACGCAAGTTTCTACCGGGCACCGTAAATGTCCGACTACAAGAAAGTTTGAATA<br>AATTTGAACGAGTTGAAAAGGACAAGTTCTTTTCTGTTTGCTCTTATTTTTCACA<br>CTTTCTGCACTTCCAGAATTTGTGAAGGATAAGAGCTTTTTTTGTTTACTAGTAC<br>ATTTAAGTAAAGGAGTTTGTTATGACCATGATTACGCCAAGCTTGCATGCCTGC<br>AGGTCGACTCTAGAGGATCCCCGGGTACCGAATTCTAATA
